# Showing—intentional communication—in dogs (*Canis familiaris*)

**DOI:** 10.3389/fpsyg.2025.1608797

**Published:** 2025-06-25

**Authors:** Marianne T. E. Heberlein, Lina V. Oberliessen, Zsófia Virányi, Christiane Lutonsky, Dennis C. Turner

**Affiliations:** ^1^Core Facility Wolf Science Center, University of Veterinary Medicine Vienna, Vienna, Austria; ^2^Animal Behavior, Department of Evolutionary Biology and Environmental Studies, University of Zurich, Zürich, Switzerland; ^3^Messerli Research Institute of Human-Animal Interactions, University of Veterinary Medicine Vienna, Medical University of Vienna and University of Vienna, Vienna, Austria; ^4^Section of Physical Therapy, Clinical Department for Small Animals and Horses, Clinical Centre for Small Animal Health and Research, Small Animal Surgery, University of Veterinary Medicine, Vienna, Austria; ^5^I.E.T./I.E.A.P., Horgen, Switzerland

**Keywords:** *Canis familiaris*, cognition, intentional communication, dog, gaze alternation, intentional, referential, showing

## Abstract

**Introduction:**

The characterization of imperative pointing as intentional communication, aimed at eliciting specific actions from a partner, has been debated, with some suggesting it reflects an understanding of others as causal agents rather than attributing intentional states to them. While gaze alternation has been identified as an important form of intentional communication in humans and apes, its interpretation in dogs remains unclear.

**Methods:**

This research investigates dogs’ capacity for gaze alternation and other showing behaviors, examining their flexibility in adjusting to the cooperativeness or knowledge state of their human partners. Two experiments were conducted: (1) hiding food in the presence of dogs either with or without their owners observing the hiding procedure, and (2) hiding food in the presence of dogs and a cooperative or a competitive human partner. In the first experiment the behaviors of 21 dogs and in the second experiment 23 dogs were analyzed.

**Results:**

Dogs exhibited more gaze alternation and food-directed showing behaviors when their owner lacked knowledge of the food location and in the presence of a cooperative partner. Conversely, they showed an empty hiding place to the competitive partner, suggesting an understanding of the partner’s intention to consume the hidden reward.

**Discussion:**

In the two independent experiments, we showed how flexibly dogs adapt their showing behavior to the knowledge or expected behavior of their human partners. These findings confirm dogs’ comprehension of the informative value of their behavior, suggesting that their showing behavior is a form of intentional communication.

## 1 Introduction

Intentional communication is a fundamental aspect of human social interactions, facilitating the exchange of information, intentions, and desires (Lyons, 1972; Woodruff and Premack, 1979). In human society, intentional communication shapes social dynamics through object-directed behaviors and social cues (Harding and Golinkoff, 1979; Bard, 1992). Even in its simplest forms, such as declarative pointing observed in human infants, intentional communication transcends linguistic boundaries (Bates et al., 1975; Liszkowski et al., 2006). This behavior prompts an exploration into the evolutionary roots of intentional communication, extending to non-human animals such as chimpanzees, orangutans, gorillas, and bonobos, which exhibit pointing gestures accompanied by gaze alternation (Harding and Golinkoff, 1979; Leavens and Hopkins, 1998; Leavens et al., 2005).

**Figure 1:**
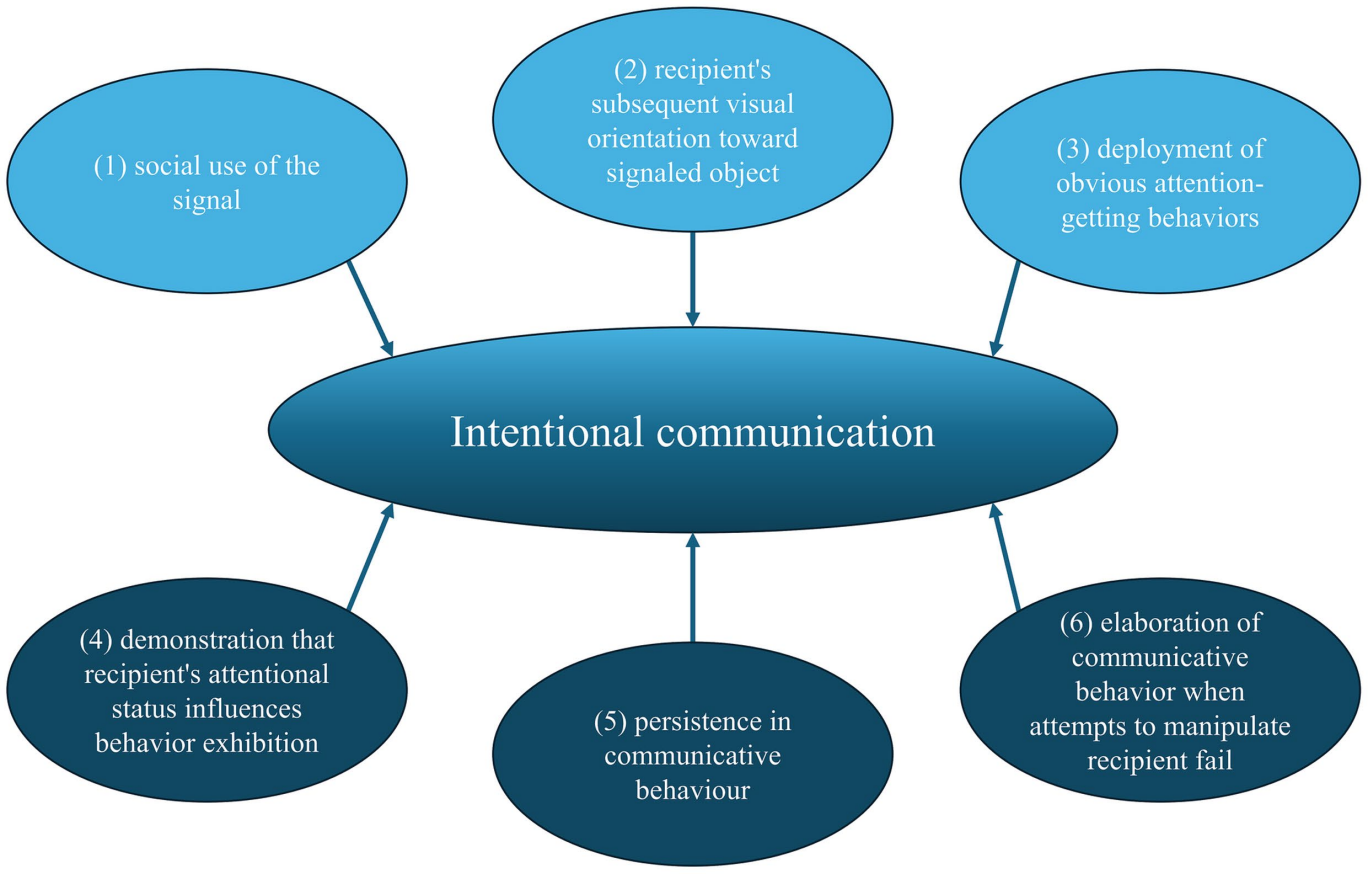
Criteria to declare a signal as a form of intentional communication as defined by Leavens et al. (2005).

Non-human studies often employ behavioral criteria (Figure 1) to define intentional communication (Leavens et al., 2005; Franco and Butterworth, 1990; Franco and Butterworth, 1996; Tomasello and Camaioni, 1997; Golinkoff, 1986; Bates et al., 1975).

Pointing and gaze alternation in apes, particularly chimpanzees, fulfill these criteria (Leavens et al., 1996; Leavens et al., 2005; Leavens and Hopkins, 1998; Hostetter et al., 2001). Dogs also exhibit intentional communication, meeting criteria such as social use in the presence of an audience, the recipient’s visual orientation, and attention-seeking behaviors (Miklósi et al., 2000; Gaunet and Deputte, 2011). Although dogs demonstrate persistence, their behavior lacks elaboration.

This pattern suggests a sophisticated integration of object-and partner-directed behaviors, possibly driven by conditioning rather than by an attempt to influence the receiver’s intentions (Leavens et al., 2005). However, gaze alternations and directional components toward a hidden food reward and an owner unaware of its location are present in 4–6-month-old puppies to an extent that is, surprisingly, not different from that of dogs aged between 2 and 11 years (Prato-Previde et al., 2023). Therefore, conditioning cannot be considered the sole origin of such behavior.

To date, few studies have addressed the question of to what extent gaze alternation and other “showing” behaviors in dogs aim at influencing their partner’s mental states or simply their behavior (e.g., to get them from one location to another) without taking their internal state (e.g., knowledge, attention) into account (Camaioni, 1993). The majority of studies on pet dogs (Miklósi et al., 2000; Gaunet, 2010; Gaunet and Deputte, 2011) used a reward hiding (“showing”) paradigm to examine whether and when dogs indicate to their human partners where food or a toy, placed out of dogs’ reach, can be found. Given that the animals’ only chance to get access to the reward is asking their human partner to give it to them, showing behavior can easily be interpreted as an attempt to influence the human’s behavior: to direct them to a certain location and to make them get the reward for the subject. Further sophistication of the procedure and comparison of different conditions are needed to investigate whether dogs also take their partner’s mental states (ignorance or intentions) or at least the contextual factors or humans’ former behaviors indicative of these mental states into account.

Virányi et al. (2006) conducted a study to investigate whether dogs take their owner’s previous presence or absence into account when requesting their favorite toy. They found that pet dogs indicated the location of their toy more often when their owner did not know where the reward was hidden, compared to when their owner was knowledgeable. However, because only in the knowledgeable condition had the owners been involved in playing with the toy and hiding it, the authors suggested that these results might be explained by dogs simply intensifying their showing behavior after their owner’s previous disengagement from an exciting event in the ignorant condition (Virányi et al., 2006). By using another paradigm, Kaminski et al. (2011) aimed to investigate whether dogs take others’ intentions into account and provide humans with specific information about the location of an object needed by the person. The dogs showed their owners where human-used—but dog-uninteresting—objects were located, yet they did not distinguish between objects their owners needed and those they did not. Therefore, the authors suggested that the dogs would rather execute their owners’ directives and requests than inform them (Kaminski et al., 2011).

Given these limited results, the present study aimed to further investigate how flexible dogs adjust their behavior in response to the knowledge states and intentions of their human partners. In the first experiment, we aimed to re-examine the effect found by Virányi et al. (2006). We tested dogs with an informed versus an uninformed owner; however, the owner was never directly involved in the hiding procedure, and the two conditions differed only in the passive presence (Knowledgeable owner condition) or absence of the owner (Ignorant owner condition) while another person hid the food. In the second experiment, we confronted the dogs with a “Cooperative” and a “Competitive” partner. The so-called “Cooperative” Partner always gave the food reward to the dog, while the “Competitive” one ate the food herself. To minimize learning effects that can explain the different responses of the dogs towards these two partners, the dogs were tested with two unfamiliar experimenters whose role they had learned in a setup different from the later context of the showing test. In the showing test, we analyzed whether the dogs indicated the food location to the “Cooperative” partner more often than to the “Competitive” one, and whether they attempted to mislead or confuse the Competitive Partner more often than the Cooperative one.

## 2 Experiment 1: knowledgeable vs. ignorant owner

### 2.1 Materials and methods

#### 2.1.1 Subjects

A total of 21 dogs (M/F: 8/13) of different breeds and mongrels participated with their owners (all women) in this experiment. All dogs were between 1 and 14 years of age (mean: 7.90; SD: 3.30) and were owned by private individuals.

#### 2.1.2 General experimental setup

**Figure 2:**
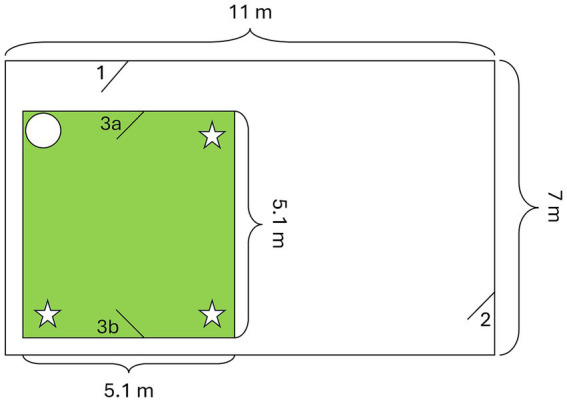
Layout of the testing room (green square). The potential food locations, hanging from the ceiling at a height of 170 cm, are marked with stars, and the circle indicates the position of a chair. (1) & (2) Entrances to the enclosure, entrance (1) was used by the dogs and their owners. (3a) The dogs and their owners entered and left the room through this door. (3b) The experimenter entered and left the room through this door.

The experiment was conducted outdoors in a secluded enclosure at the University of Zurich-Irchel, Switzerland. The enclosure was surrounded by concrete walls on three sides, and on the remaining side, there was an empty enclosure separated by a high fence covered with a bamboo screen. The entrance was a gridded door covered with opaque plastic sheets. Inside the enclosure, an inner section was built with opaque plastic sheets, which served as the effective test area (Figure 2). This test area featured two opposing doors and a chair in one corner, while each of the other three corners housed a box suspended from the roof at a height of 170 cm, making them inaccessible to the dogs but accessible to a human for food retrieval. These boxes served as hiding places during the experiment.

#### 2.1.3 Procedure

Before the experiment began, the dogs had the opportunity to explore the test area for a few minutes, the duration of which depended on the dog’s level of excitement. The more excited the dog was, the more time it needed. Afterwards, a pre-feeding was conducted.

#### 2.1.4 Pre-feeding

The pre-feeding, as performed in a previous study (Heberlein et al., 2016), aimed to familiarize dogs with the concept that three boxes could contain food, and their owner was willing to provide them with this out-of-reach reward. The owner baited all three boxes with sausage, ensuring the dog paid attention to the hiding procedure. The order of baiting was predetermined and randomized across dogs and trials. Subsequently, the owner stood in the test area, asking the dog to identify the box with food. If the dog looked at a box, the owner would reward it by giving the dog food from that box. This process continued until all the boxes were emptied. If a dog consistently indicated an empty location, the owner showed her an empty hand after reaching into the box. If a dog failed to look at any box, the owner approached a random box and waited for the dog’s attention before providing the reward. The hiding and showing procedure persisted until, over at least two consecutive trials, the dog looked at all boxes without extensive prompting.

#### 2.1.5 Test

In the test phase, directly after the pre-feeding, the experimenter hid a piece of sausage in one of the three boxes while making sure that the dog paid attention (called the dog’s name when the dog was not observing the hiding). The owner was either present (Knowledgeable owner condition) or absent (Ignorant owner condition). In the “Knowledgeable owner condition,” the owner observed the hiding procedure while standing silently at the entrance of the testing room (3a see Figure 2). However, during the hiding in the “Ignorant owner condition,” the owner was waiting outside of the test enclosure unable to see the hiding (behind door 1 see Figure 2). The dogs observed the owners leaving the enclosure. After hiding the food, the experimenter left the test room, and in the “Ignorant owner condition,” the owner entered the enclosure. In all conditions, the owner closed the test room and sat down in the chair. The owner remained passive, neither talking to nor touching the dog but observing it for 1 min. After this, the owner attempted to find the hidden sausage based on the dog’s behavior and her knowledge. In case the owner found food in the chosen box, the dog received the hidden sausage; if the box was empty, she presented her empty hand to the dog after reaching into the box.

Four test trials were conducted in succession: in two trials, the owner was absent during the hiding period, and in the other two trials, the owner was present. The order of trials was predetermined and semi-randomized across dogs, with the stipulation that the first two trials did not belong to the same condition. The food locations were predetermined and semi-randomly assigned across dogs and trials, ensuring that each location was used at least once, and the fourth one was chosen randomly.

#### 2.1.6 Analysis

The experiment was videotaped with a digital video camera (JVC GZ-MG330HE). During the 1 min when the owner was passive, we analyzed the dog’s behavior and measured the behaviors listed in Table 1.

**Table 1:** Ethogram of the analyzed behaviors.

Behavioral category	Behavior	Description of the behavior
showing behavior*	Gaze alternation*	Looking at the owner/partner’s face and then directly (with continuous head movement without interruption) at one of the boxes or vice versa
	“Other showing behaviors”*	Going to the owner/partner, looking at them or touching them with the nose, and then going to one of the three boxes and looking at it without stopping in between (continuous movement)
		Going to the owner/partner, looking at them or touching them with the nose, and then looking at a box (continuous movement)
		Looking at the owner’s face and then going to a box and looking at it without stopping in between (continuous movement)
	Looking at a potential food location*	The dog’s head is pointing towards a potential food location. This behavior is only coded if it is not part of the behavioral chain described as “showing behavior.”
	Duration of looking at the partner/owner	The dog’s head is pointing towards the owner/partner’s face. This behavior is only coded if it is not part of the behavioral chain described as “showing behavior.”

*Each of these behaviors was coded separately for the food location and the empty locations.

#### 2.1.7 Statistical analysis

We calculated generalized linear mixed effects models using a Poisson distribution to investigate the influence of age, sex, condition (knowledgeable or ignorant owner), and serial number of trials on the frequency of gazing and showing behaviors. The individual and the hiding location were included in the model as random factors. The time the dogs spent looking at their owner was analyzed using a linear mixed-effects model with the same fixed and random factors described above. To obtain a normal distribution of the residuals, we applied a square root transformation.

To assess inter-observer reliability, 34.1% of the videos were analyzed by a second person who was blind to the test condition, the hiding location, and the purpose of the test. Spearman’s rank correlations (rho) between the two coders were, in general, high: Duration of looking at owner: 0.96; Frequency of looking at food location: 0.85; Frequency of looking at empty locations: 0.81; Frequency of showing food location: 0.82; Frequency of showing empty locations: 0.84. The analyses were conducted using R 2.15.2 (R Core Team, 2012).

### 2.2 Results

**Figure 3:**
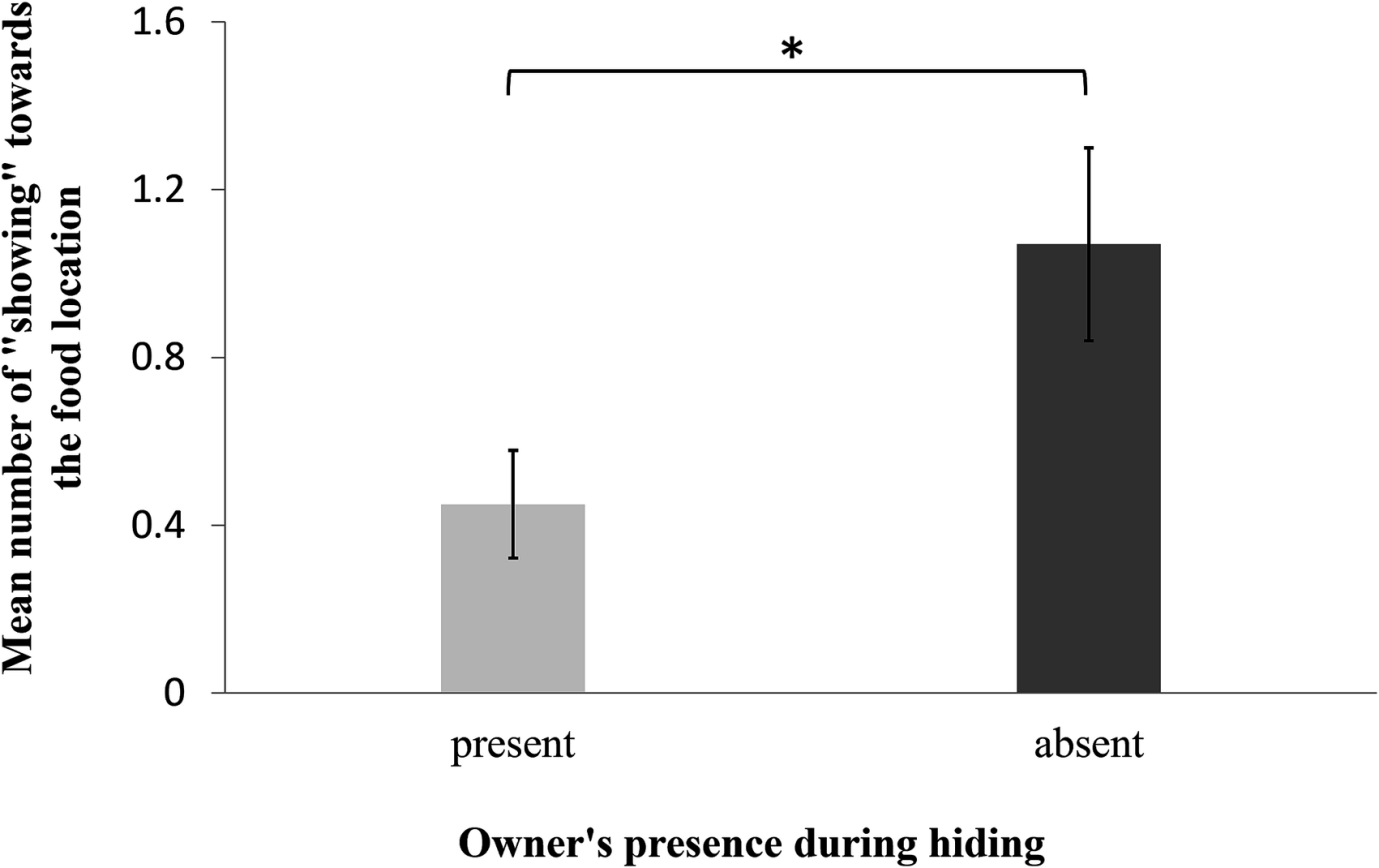
Mean number of showing behaviors toward the food location over all sessions in the presence of the owner during the “Knowledgeable” vs. “Ignorant owner condition”.

Dogs displayed more showing behaviors towards the food location if the owner had been absent during food hiding than when she had been present (GLMM: F_1,62_ = 6.010, *p* = 0.007; Figure 3). Age, sex, and trial number did not influence the occurrence of showing (GLMM: age: F_1,18_ = 0.02, *p* = 0.90; sex: F_1,19_ = 0.23, *p* = 0.64; trial: F_4,62_ = 0.92, *p* = 0.46). Furthermore, the dogs showed their owners the empty food locations similarly often in both conditions (GLMM: F_1,62_ = 1.39, *p* = 0.20). Age, sex, and trial number did not influence the occurrence of showing the empty boxes either (GLMM: age: F_1,18_ = 0.77, *p* = 0.40; sex: F_1,19_ = 0.90, *p* = 0.40; trial: F_1,60_ = 0.69, *p* = 0.60).

In contrast to showing behaviors, the dogs looked similarly often at the food location independently of whether the owner had been present or absent during hiding (GLMM: F_1,62_ = 1.65, *p* = 0.20). Age, sex, and trial had no influence on the number of looks at the baited box (GLMM: age: F_1,19_ = 0.69, *p* = 0.40; sex: F_1,18_ = 0.10, *p* = 0.80; trial: F_4,61_ = 0.50, *p* = 0.70). Age, sex, and trial number had no influence on the number of looks the dogs directed at the empty boxes (GLMM: age: F_1,17_ = 0.03, *p* = 0.86; sex: F_1,19_ = 0.91, *p* = 0.35; trial: F_4,58_ = 1.23, *p* = 0.31). However, the dogs looked more often at the empty locations when the owner was present during food hiding (GLMM: F_1,62_ = 7.240, *p* = 0.009). Furthermore, dogs looked longer at the owner if she had been absent during food hiding (lme: F_1,62_ = 15.600, *p* < 0.001). Again, we found no influence of age, sex, and trial number on this variable (lme: age: F_1,19_ = 2.54, *p* = 0.10; sex: F_1,18_ = 0.02, *p* = 0.90; trial: F_4,60_ = 0.11, *p* > 0.99).

The differences between the two conditions most likely were not driven by higher arousal in the dogs after their owner’s absence, because the frequency of looking at all three boxes did not differ between the two conditions (GLMM: F_1,62_ = 0.86, *p* = 0.40). Age, sex, and trial number had no influence on this variable either (GLMM: age: F_1,19_ = 0.28, *p* = 0.60; sex: F_1,18_ = 0.10, *p* = 0.80; trial: F_4,59_ = 0.49, *p* = 0.70).

Summarizing the results, we found that the dogs differentiated between the “Knowledgeable” and “Ignorant owner conditions”: they showed the food location more often when the owner had been absent during food hiding compared to when the owner had been present, they looked at the two empty boxes more often after the owner had been present during food hiding and they looked longer at the owner when she had been absent during the food hiding. At the same time, the sum frequency of looking at all boxes did not differ across conditions, indicating that the above-mentioned differences should not be explained by the higher arousal of the dogs after their owner’s absence.

## 3 Experiment 2: cooperative vs. competitive partner

In the second experiment, we tested the dogs in a context that dogs rarely encounter: they had to differentiate between two persons who had either given them food or eaten it themselves.

### 3.1 Materials and methods

#### 3.1.1 Subjects

A total of 23 domestic dogs (M/F: 7/16) of different breeds and mongrels participated in this experiment along with their owners. Two owners (one with four dogs and one with two dogs) were unable to take part in the third test day. All dogs were between 0.75 and 14 years of age (mean: 7.3; SD: 3.6) and were owned by private individuals. The majority of dogs (70%) lived with at least one other dog in the same household. The test area remained the same as in Experiment 1 (Figure 2).

#### 3.1.2 Procedure

The experiment spanned a minimum of 4 days, with the exact duration determined by how quickly each dog met the preference-test criterion (Table 2).

**Table 2:** Experimental procedure.

Day 1	Day 2	Day 3
Pre-feeding	Pre-feeding	Preference test
↓	↓	↓
Training (3×2)	Training (3×2)	Test (2×2)
↓	↓	
Break (at least 3 min)	Preference test	
↓	↓	
Training (3×2)	Test (2×2)	
↓		
Preference test		

#### 3.1.3 Pre-feeding

The owner conducted the pre-feeding in the same way as described in Experiment 1.

#### 3.1.4 Training

The first 2 days involved pre-feeding, followed by a training session (Table 2) where dogs were acquainted with the roles of two human partners. Both partners were women of a similar age. The “Cooperative Partner” consistently rewarded the dog, while the “Competitive Partner” ate the food herself. For 15 dogs, the Cooperative Partner was completely unknown, whereas they had met the Competitive Partner a few times before the test. Thus, if at all, the former experiences of the dogs with this person were likely to influence their behavior in contrast to our predictions. For the remaining eight dogs, both partners were unfamiliar, allowing statistical testing for the competitive partner’s familiarity effect. During training, dogs were leashed and positioned on one side, with a bowl of sausages placed about two meters away. Partners, in a predetermined order, approached the bowl three times and, after calling the dog’s name, either ate the sausage or gave it to the dog. This procedure was repeated on the first and second test days after a break of at least 3 min.

#### 3.1.5 Preference test

We investigated whether dogs learned the roles of their two partners prior to each test. The preference test involved the owner holding the dog on one side of the testing room while the two partners stood 3 meters apart on the opposite side. Both partners simultaneously presented a piece of sausage to the dog, and the owner released the dog to approach one of them. If the dog chose the Cooperative Partner, it could eat the sausage, but if it chose the Competitive Partner, the person ate the sausage demonstratively before the dog could reach it. After each choice, the owner called the dog back. Four consecutive trials were conducted, with predetermined and semi-randomized positions for the partners. Preference tests were performed at the end of the first day and after training on the second day. Dogs choosing the Cooperative Partner in at least 3 out of 4 trials on the second day proceeded to the test. If not, the preference test was repeated after a 3-min break, and if unsuccessful again, a third attempt was made. Ten dogs (43.48%) reached the criterion in the first preference test on the second day, 9 (39.13%) in the second test, and four dogs (17.39%) needed three preference tests. None of the dogs failed all three tests on the second day. On the third day, another preference test was conducted, and all dogs successfully met the criterion to participate in the second test.

#### 3.1.6 Test

The owner and dog entered a testing room where the owner hid a piece of sausage in one of three boxes in the absence of the partners, making sure that the dog observed the process. The owner then left the enclosure, and one of the two partners entered. They sat down on the chair and called the dog’s name once. They then observed the dog for 1 min without moving or talking. Afterward, they checked the box where they believed the food was. If the sausage was found, the Cooperative Partner gave it to the dog; the Competitive Partner ate it themselves. If the box was empty, the partner showed their empty hand. This process was repeated for four trials, with dogs tested twice with each partner in a random order but without repetition with the same partner in the first two trials.

#### 3.1.7 Behavioral coding and statistical analysis

The experiment was videotaped with a digital video camera (JVC GZ-MG330HE), and the same behaviors were coded as in Experiment 1 (see Table 1). However, in addition to age and sex, we included partner present and test day as fixed factors in the models. Furthermore, we included the familiarity of the Competitive Partner to check whether it had an effect. However, since we did not find any difference between the familiarity and unfamiliarity of the Competitive Partner, we no longer mention this in the results section.

In addition, regarding each dog that showed an empty location to the Competitive Partner, we further investigated whether its behavior was suitable to mislead the Competitive Partner. For this aim, we categorized a dog as a “Misleader” if, in a trial, it showed only one empty food location or showed one empty location more often than another location (in contrast to other dogs that showed each of two or three food locations with the same frequency or showed the actual food location more often than the empty ones). With a one-sample t-test, we investigated whether most of the dogs that showed an empty location to the Competitive Partner were “Misleaders.” The same categorization of dogs and analysis of the number of “Misleaders” was also applied to dogs that simply looked at an empty location.

Inter-observer reliability tests were conducted with Spearman’s rank correlation. 60.6% of the videos were analyzed by a second person blind to the purpose of the test as well as the condition (cooperativeness of the partner) and the hiding place in each trial. Spearman’s rank correlations (rho) were in general high: Duration of looking at partner: 0.78; Frequency of looking at food location: 0.84; Frequency of looking at empty locations: 0.77; Showing behavior: food location: 0.80; empty location: 0.87. The analyses were performed using R 2.15.2 (R Core Team, 2012).

### 3.2 Results

**Figure 4:**
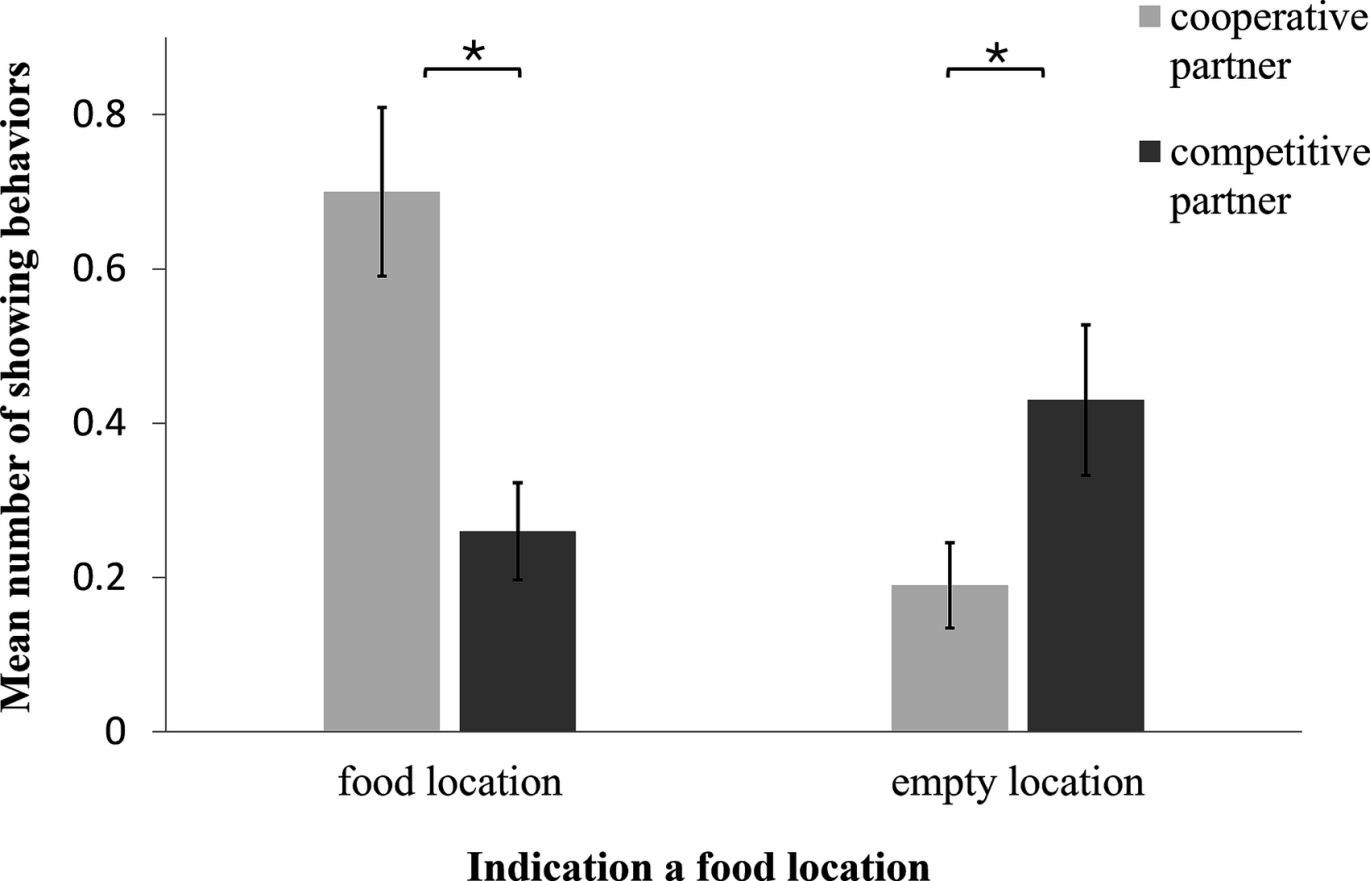
Mean number of showing behaviors over all sessions towards a potential food location in the presence of the cooperative vs. competitive partner. The dogs indicated the food location more often in the presence of the cooperative compared to the competitive partner. However, they indicated an empty food location more often to a competitive partner than to a cooperative partner.

We recorded more showing behaviors toward the food location in presence of the Cooperative Partner than when a Competitive partner was present (GLMM: F_1,140_ = 14.900, *p* < 0.001; Figure 4). In addition, in the presence of the Competitive Partner, more showing behavior occurred toward the two empty boxes than in the presence of the Cooperative Partner (GLMM: F_1,162_ = 4.590, *p* = 0.030; Figure 3). In the case of neither of these two variables did we find an influence of age, sex, trial number or test day (GLMM: indicating food location: age: F_1,21_ = 1.58, *p* = 0.20; sex: F_1,21_ = 0.99, *p* = 0.30; trial: F_5,137_ = 0.18, *p* > 0.99; test day: F_1,151_ = 1.15, *p* = 0.30; indicating empty box: age: F_1,161_ = 1.74, *p* = 0.19; sex: F_1,160_ = 1.72, *p* = 0.19; trial: F_5,154_ = 0.14, *p* = 0.98; test day: F_1,159_ = 0.58, *p* = 0.45). Upon examining the behavior of the dogs that indicated an empty food location to the Competitive Partner, we found that the majority of them did so in a way that could mislead the Competitive Partner (one-sample t-test: t = 2.621, *p* = 0.021). Specifically, among the dogs that indicated an empty location, more dogs than could be expected by chance focused on a single empty location or showed an empty location more often than the other two locations.

**Figure 5:**
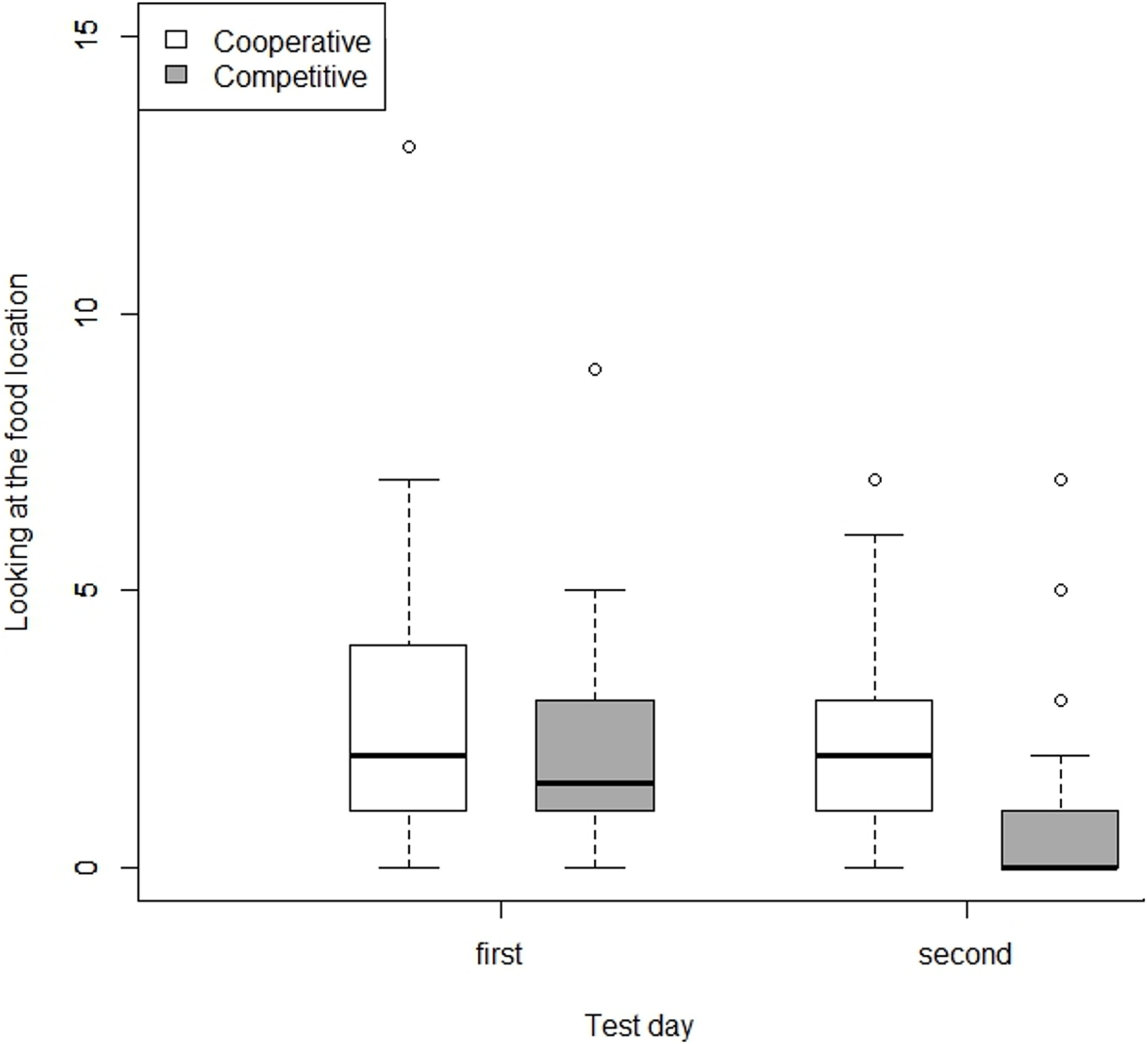
Number of looking at the food location per test day in the presence of cooperative and competitive partners. While we found no difference between the two partners on the first test day, we found a decrease in looking at the food location during the second test day with the competitive partner.

In contrast, we did not find the same effect of partners on the number of looks directed at the food location in the first test day: there was no difference in the presence of the Cooperative and the Competitive Partner (GLMM: F_1,70_ = 0.37, *p* = 0.55). There was a near-significant trend for male dogs to look at the food location more frequently than female dogs (GLMM: F_1,21_ = 4.300, *p* = 0.051). There was no effect of age and trial number on this variable (GLMM: age: F_1,20_ = 0.60, *p* = 0.45; trial: F_4,68_ = 0.24, *p* = 0.91). On the second test day, however, the dogs looked more often at the food location in the presence of the Cooperative Partner (GLMM: F_1,50_ = 16.600, *p* < 0.001). Importantly, this effect seems to be because the dogs, specifically in the presence of the Competitive Partner, showed this behavior less frequently on the second test day than on the first day (Figure 5). We found no influence of age, sex, and trial number on the second day (GLMM: age: F_1,15_ = 0.36, *p* = 0.60; sex: F_1,15_ = 0.47, *p* = 0. 50; trial: F_5,48_ = 0.29, *p* = 0.90).

When with the Competitive Partner, the dogs looked more often at empty boxes, unlike when with the Cooperative Partner (GLMM: F_1,141_ = 5.210, *p* = 0.020). We found no influence of age, sex, test day, and trial number (GLMM: age: F_1,21_ = 0.78, *p* = 0.39; sex: F_1,21_ = 0.82, *p* = 0.37; test day: F_1,152_ = 2.16, *p* = 0.14; trial: F_5,137_ = 1.46, *p* = 0.21). In contrast to showing an empty location, the dogs did not characteristically look at a specific empty box in a misleading way. A similar number of dogs focused on a single empty box or looked at multiple boxes (one-sample t-test: t = −0.37, *p* = 0.72).

At the same time, the frequency of looking at all three boxes did not differ between the Cooperative and Competitive Partner conditions (GLMM: F_1,135_ = 0.06, *p* = 0.80), indicating that the dogs were not more aroused in the presence of either Partner. Rather, in both cases, on the first test day, the dogs looked at the three boxes more often than on the second day (GLMM: F_1,149_ = 10.800, *p* = 0.001). We found no influence of age, sex, or trial number on this variable either (GLMM: age: F_1,21_ = 0.68, *p* = 0.40; sex: F_1,21_ = 2.24, *p* = 0.15; trial: F_5,137_ = 1.08, *p* = 0.40).

Analyzing the duration the dogs spent looking at their partners, we found an interaction between partner and test day (lme: F_1,136_ = 7.875, *p* = 0.006). On the first day, the dogs looked at both partners for similar durations (lme: F_1,69_ = 0.144, *p* = 0.70); however, on the second day, they spent more time looking at the Cooperative Partner than at the Competitive one (lme: F_1,50_ = 12.229, *p* < 0.001). We found no influence of age, sex, and trial number (lme: age: F_1,20_ < 0.01, *p* = 0.96; sex: F_1,20_ < 0.01, *p* = 0.95; trial: F_5,137_ = 1.07, *p* = 0.38).

Overall, we found that the dogs differentiated between the Cooperative and Competitive Partner conditions: they showed the baited location more often to the Cooperative Partner than to the Competitive one, whereas they showed an empty location to the Competitive Partner more often than to the Cooperative one. Importantly, they did this in a specific way (focusing on one empty location) that could mislead the Competitive partner. Furthermore, although on the first test day, the dogs often looked similarly at the food location in the presence of either partner, by the second test day, they appeared to have learned to inhibit this behavior in the presence of the Competitive Partner. They also looked at the empty locations more frequently in the presence of the Competitive Partner than in the presence of the Cooperative Partner. However, they did not focus on looking at one empty location. This was in contrast to the behavior of showing an empty food location. Finally, on the second test day, the dogs spent more time watching the Cooperative Partner than the Competitive one.

## 4 Discussion

In two independent experiments, we demonstrated how flexible dogs adapt their showing behavior to the knowledge or expected behavior of their human partners. In the first experiment, we confirmed the effect found by Virányi et al. (2006) in a more rigorous setting: dogs informed their ignorant owner about the location of a food reward more often than their knowledgeable owner. Importantly, in our study, the owner was never directly involved in the hiding procedure, and the two conditions differed only in her passive presence or absence while another person hid the food. In the second experiment, we found that dogs more often indicated where food was hidden to a Cooperative Partner than to a Competitive one, whereas they more often indicated an empty location to the Competitive than to the Cooperative one. Notably, the dogs even focused their showing of an empty location on one of these locations, thereby functionally misleading the Competitive Partner. Importantly, the dogs had learned the predictable response of these two partners to finding food in a different context than the test itself. In this different training setting, the Cooperative Partner always gives the dog a food reward after retrieving it from a food bowl. In contrast, the Competitive Partner had always eaten the food herself.

The first question to ask is whether these results can be explained by dogs being more aroused and, therefore, more active after the return of their owner and in the presence of a cooperative human partner [arousal hypothesis (Kaminski et al., 2011)]. Since dogs are likely to greet their returning owner and a person who has rewarded them on previous occasions, they are likely to exhibit increased arousal under these conditions. Increased arousal may then lead to increased activity in general and may increase the frequency with which dogs look at the food location, as well as their partner (Miklósi et al., 2000). If this is the case, the frequency of showing may also increase. If the dogs had looked at the food location and their partner more often in the Ignorant Owner and Cooperative Partner conditions, the amount of showing might have increased in these conditions simply because these two behavioral components would have had a higher probability of occurring together by chance. However, we refute the arousal hypothesis on three grounds: (1) When tested with a Cooperative vs. Competitive Partner, on the first test day, the difference between conditions was apparent in showing behavior but not in the number of looks to the food location, (2) The dogs looked for similar durations at the cooperative and the competitive partners on the first test day, although on the second test day the dogs looked longer at the cooperative partner than at the competitive one and they looked longer at the ignorant owner than at the knowledgeable one, and most importantly, (3) This version of the arousal hypothesis cannot explain why dogs showed the empty locations more often to the competitive partner than to the cooperative one.

Another potential explanation is that showing behavior is influenced by conditioning during everyday experiences (Virányi et al., 2006). Dogs may have learned through experience that communicating the location of a lost item is more necessary when the owner is absent during the hiding process. Although age did not show significant effects, these rules may be learned early in a dog’s life. However, previous research found no significant differences in gaze alternations and directional components to a hidden food reward and an owner unaware of its location between puppies and adult dogs (Prato-Previde et al., 2023). Further, this explanation is less applicable to our Experiment 2, where dogs interacted with two unfamiliar partners. First, the results cannot be explained by conditioning during the experiment since the training (learning about the cooperative and competitive character of the two partners) took place in a different context than the subsequent test. Second, even lifelong experience cannot explain the difference in the behavior of showing a food location or an empty location between the two partners. It is unlikely that dogs have ever experienced a similar situation where an unfamiliar human demonstratively ate the food after a dog requested it. Interestingly, the dogs appeared to adapt instantaneously to the novelty of interacting with a competitive human since, from the first test trial, they indicated the food location more often to the Cooperative Partner and, importantly, pointed to an empty location more often to the Competitive Partner, supporting the interpretation that dogs formed an immediate understanding of the partners’ intentions or expected behaviors during the experiment.

Dogs not only reduced the behavior of showing the food location to a Competitive Partner but also showed one of the empty locations in a misleading way. The use of tactical deception in dogs against humans was demonstrated in a previous study, where dogs were confronted with a cooperative and competitive human partner (Heberlein et al., 2017a). Animals that engage in tactical deception, a form of behavioral deception, must have some understanding of how their deceptive actions affect the behavior of other individuals (Santos et al., 2006). It has been defined that behavioral deception involves the use of false signals to alter the behavior of a receiver, resulting in a benefit to the sender and a cost to the receiver. The costs can be very high, such as the loss of life, or relatively low, such as the expenditure of energy to find another foraging site or, in our case, to relocate to an empty food location (Semple and McComb, 1996). However, indicating an empty food location to the Competitive Partner in the present study does not result in a benefit in terms of food. Regardless of whether they show the correct location, an empty location, or no location, the dogs do not receive any food reward in the Competitive Partner condition since neither the cooperative partner nor the owner provides them with the leftover food piece. While the dogs may have expected to receive the leftover food piece from the owner after the Competitive Partner left the test area during the first or even second test trial, we do not expect them to have this expectation later. The value of showing the empty food location could, however, be due to dogs’ inequity aversion. Numerous studies have reported dogs’ aversion to unequal reward distributions that favor another individual, known as disadvantageous inequity aversion [for a review, see McGetrick and Range (2018)]. In the current experimental scenario, by indicating an empty food location, the dogs prevent the Competitive Partner from obtaining the reward, thereby maintaining a relatively better outcome for themselves.

The results of this study suggest that showing behavior goes beyond mere signaling of object location to intentional communication aimed at influencing the partner’s behavior. Dogs may possess an understanding of the informative value of their actions, as evidenced by their differential responses to knowledgeable versus ignorant owners and cooperative versus competitive partners. It remains an open question, however, to what extent this flexible signaling of dogs reflects an understanding of the human partners’ knowledge state or intentions. Even if a recent false belief study has suggested that dogs are capable of inferring human knowledge states based on the previous absence vs. presence of their human partners (Lonardo et al., 1955), in our study, as explained earlier, we cannot exclude that dogs, applying a behavioral rule acquired during their life-long experiences, adjusted their showing behavior directly to their owner’s presence/absence during a significant event. However, as discussed earlier, explaining the dogs’ flexible behavior and, especially, their misleading behavior in accordance with such a behavioral rule is much more difficult in Experiment 2. Supporting the argument that the dogs’ misleading behavior was driven by considering the intentions of their human partners, dogs have also been shown to adjust their responses to a human’s misleading signal based on that person’s level of knowledge (Lonardo et al., 1955). For contradicting results, see Lonardo et al. (2024) and Völter et al. (1991). Dogs were more likely to follow a human’s obviously misleading suggestion to choose one of two food locations when the human did not know that the location was empty than when the human suggested this location even though she knew it was empty (because she was present when the food was moved from this location to the second location). The authors of this study suggested that dogs (unlike human infants and chimpanzees) may be less likely to follow the knowledgeable person’s misleading suggestion because they interpret this behavior as deceptive or driven by an intention other than telling the dog where to find food, which the dogs were less likely to follow (Lonardo et al., 1955, p. 5). Interestingly, this study also reported that cooperative breeds seemed to drive this differentiation. In contrast, terriers seemed to have complied with such an alternative intention of the human and tended to follow her misleading cue when she knew there was no food there.

These findings suggest that different breeds may not only respond differently to deceptive behaviors but also use them in distinct ways. Although we included a wide range of breeds in this study, our sample size did not allow us to test for breed differences, despite breed differences having also been observed in human-directed behaviors, which suggests a potential influence of genetics on communicative tendencies (Passalacqua et al., 2011; Heberlein et al., 2017b). Future research should examine how breed-specific traits interact with communication strategies to provide a more comprehensive understanding of canine communication, its mechanisms, and its origins.

In conclusion, our experiments contribute to our understanding of canine communication by providing empirical evidence for the application of behavioral criteria in controlled experimental settings. By unraveling the complexities of canine communication, we gain valuable insights into the cognitive abilities and social dynamics of our canine companions, paving the way for further exploration of interspecies communication and human-animal interactions. Furthermore, these insights have practical implications for areas such as animal training, welfare, and human-animal interactions, highlighting the need for further exploration into the complexities of interspecific communication.

## Data Availability

The raw data supporting the conclusions of this article will be made available by the authors, without undue reservation.
